# Abdominal Tuberculosis Presenting With Small Bowel Obstruction: A Case Report

**DOI:** 10.7759/cureus.37459

**Published:** 2023-04-11

**Authors:** Ammara S Sahibole, Rihab Farooq, Hafsa M Ali, Syeda J Bukhari, Labib S Al Ozaibi

**Affiliations:** 1 Department of General Practice, Dubai Academic Health Corporation, Dubai, ARE; 2 Department of Medicine, Dubai Academic Health Corporation, Dubai, ARE; 3 Department of Medicine, Thumbay University Hospital, Ajman, ARE; 4 Department of Medicine, Medcare Hospital, Dubai, ARE; 5 Department of General Surgery, Rashid Hospital, Dubai, ARE

**Keywords:** small bowel obstruction, exploratory laporotomy, anti-tuberculosis therapy, abdominal tuberculosis, abdominal tuberculosis with intestinal obstruction

## Abstract

Abdominal tuberculosis (TB) is a common form of extrapulmonary TB (EXPTB). It is being reported increasingly, especially in high-burden regions of the world.

We present a case of a 37-year-old male who presented to the emergency department with clinical features suggestive of bowel obstruction. On clinical examination, the patient exhibited generalized tenderness in the abdomen. A subsequent CT scan revealed features consistent with small bowel obstruction. The patient underwent a diagnostic laparoscopy, which was converted to an exploratory laparotomy due to intraoperative findings of adhesions. Notably, there were extensive peritoneal deposits and adhesions between bowel loops. Peritoneal biopsies were obtained and subjected to the acid-fast bacillus (AFB) smear and culture, which demonstrated the growth of the Mycobacterium tuberculosis complex. As a result, the patient was initiated on antituberculous therapy.

## Introduction

Tuberculosis (TB) is a highly contagious disease that has a significant global impact, with extrapulmonary TB (EXPTB) on the rise. Abdominal TB is a common manifestation of EXPTB, caused by either ingestion of contaminated materials or by hematogenous, lymphatic, or direct spread, affecting the peritoneum, lymph nodes, gastrointestinal lumen, or solid viscera [1]. Several important risk factors, including latent TB, chronic medical conditions such as diabetes mellitus and liver cirrhosis, and immunosuppressive infections such as human immunodeficiency virus (HIV), contribute to the rising incidence and prevalence of abdominal TB in the developed world [2]. Patients with abdominal TB often present with non-specific symptoms such as abdominal pain or distension. Diagnosis can be challenging due to the various available diagnostic tests and the expertise required to interpret test results accurately. Laboratory tests may reveal non-specific results such as anemia, thrombocytosis, and leukocytosis. Specific diagnostic tests include bacteriological examinations of specimens from the potential sites of infection and CT scans, which can help identify affected sites, bowel obstruction, and strictures [2]. Medical treatment typically involves antituberculous drugs, while surgical management may be necessary for the presence of features such as bowel obstruction [3].

## Case presentation

A 37-year-old previously healthy Pakistani male presented to the emergency department with a one-day history of central abdominal pain, colicky in nature. It was associated with multiple episodes of bilious vomiting, consisting of gastric contents, and a history of constipation for five days before presentation. He did not report fever, urinary symptoms, or weight loss and had no medical or surgical history. There was no recent history of travel to his home country.

On examination, the patient was conscious, alert, and oriented; his vital signs were stable; and he did not appear clinically dehydrated. Examination of the abdomen revealed a nondistended abdomen with generalized tenderness to palpation, which was slightly more pronounced in the umbilical region and increased bowel sounds on auscultation.

Laboratory tests done in the emergency room revealed an elevated white blood cell (WBC) count of 12.7 × 10^3 ^microL^-1^ (reference range 3.6 × 10^3^ to 11 × 10^3 ^microL^-1^), a hemoglobin level of 12.9 g/dL (reference range 13.0-17.0 g/dL), and an elevated C-reactive protein (CRP) level of 8.4 mg/L (reference range <5.0 mg/L).

**Figure 1:**
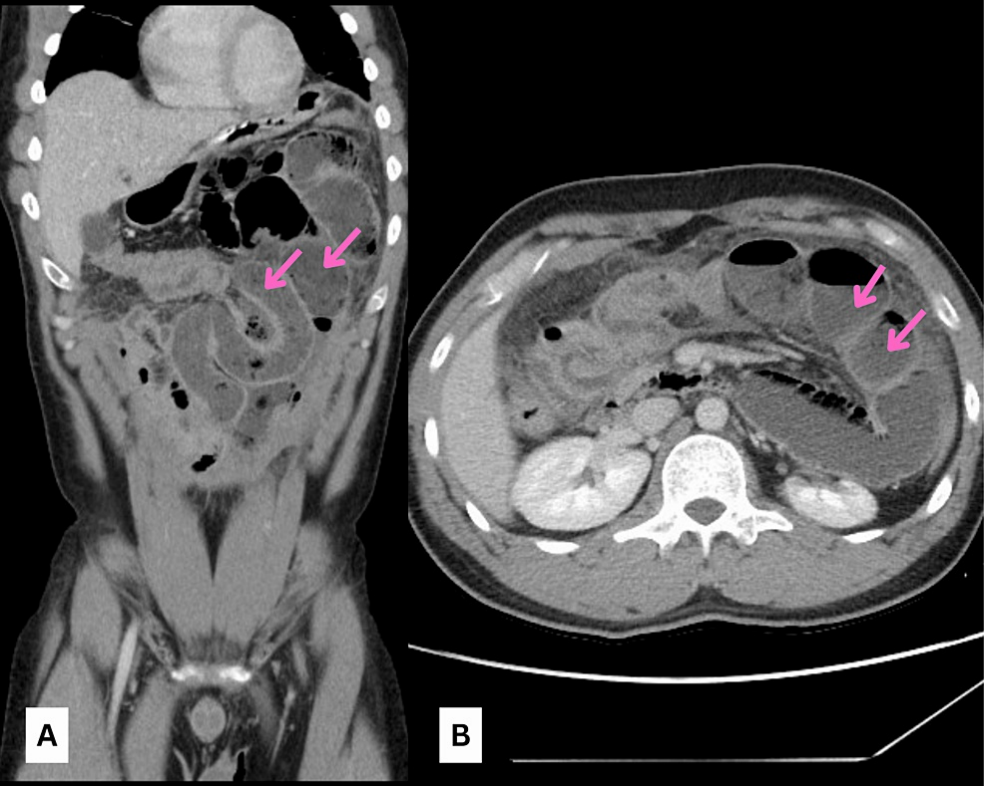
Abdominal CT scan images: (A) coronal view and (B) transverse view showing dilated jejunal loops (pink arrows). CT, computed tomography

The patient underwent a CT scan of the abdomen with contrast, which showed dilated jejunal loops on the left side of the abdomen, collapsed distal jejunal and ileal loops showing wall thickening, and mesenteric venous engorgement suspicious of inflammatory peritoneal changes. Distal ileal loops appeared normal with normal ileocecal junction, and the large bowel appeared collapsed. Minimal free fluid was seen in the pelvis (Figures 1A, 1B).

The patient was admitted under General Surgery and kept nil per os (NPO), and a nasogastric tube (NGT) was placed for decompression. He was also scheduled for further laboratory tests, namely, lactic acid, which was elevated at 2.4 mmol/L (reference range 0.5-2.2 mmol/L), and D-dimer, which was elevated at 3.27 microg/mL FEU (reference range <0.5 microg/mL FEU). He was then taken for diagnostic laparoscopy, which was converted to exploratory laparotomy due to extensive adhesions. He was found to have extensive peritoneal deposits, matted bowel, and adhesions between the bowel loops. Intraoperative biopsy was taken from the peritoneum in addition to a peritoneal fluid sample, and adhesions were released from the ileocecal valve to the ligament of Treitz.

Abdominal TB was suspected due to the presence of peritoneal deposits, and an appropriate workup was done. Abdominal fluid microscopy and culture revealed the presence of *Enterobacter cloacae* and *Klebsiella pneumoniae*, while a biopsy culture of peritoneal tissue showed no growth. Acid-fast bacillus (AFB) smear and culture of the intraperitoneal fluid showed growth of acid-fast *Mycobacterium tuberculosis* complex after 15 days. TB polymerase chain reaction (TB PCR) direct detection for biopsy detected *M. tuberculosis* complex, not resistant to rifampicin. A Quantiferon TB gold test resulted positive.

The patient was diagnosed with abdominal TB with a secondary bacterial infection with *E. cloacae*.

Postoperatively, he was referred to the infectious diseases unit (IDU) for the management of TB and was started on antituberculous therapy (ATT) for 30 days, which consisted of rifampicin-isoniazid 300-150 mg, pyrazinamide 2,000 mg, and ethambutol 1,200 mg. He was also started on amikacin 1,000 mg intravenously (IV) for the duration of the hospital stay. The patient was doing well and was discharged on postoperative day nine vitally and clinically stable on ATT.

## Discussion

TB continues to be a cause of significant health concern worldwide, affecting both developed and developing countries, with factors such as migration and immunosuppression contributing to the problem. In 2021, an estimated global total of 10.6 million people contracted TB, with a 3.6% increase in incidence rate from the previous year [4]. EXPTB accounts for 8% to 24% of total TB cases globally, with an average of 15%. High TB-burden countries such as India and Pakistan have a higher prevalence of EXPTB, with 20% and 21% of cases, respectively, accounting for abdominal TB. In medium-burden countries, the abdomen is the sixth most common site of EXPTB, accounting for 9% of cases. In low-burden regions such as the United States and Europe, the prevalence of abdominal TB is lower than in higher-burden areas, with EXPTB accounting for 20% and 17% of all TB cases, respectively, and abdominal TB accounting for 6% and 3% of cases, respectively [5].

Abdominal TB can occur through several possible routes of infection. It can occur through ingestion of contaminated materials such as infected sputum or milk, hematogenous spread from a primary focus, direct spread from an adjacent focus, or lymphatic vessels [1]. When it occurs through ingestion, the causative organism upon reaching the bowel is captured in the Peyer's patches and transported by macrophages to the mesenteric lymph nodes, where it may remain in a dormant state. Inflammatory enlargement of Peyer's patches causes mucosal ulcerations and eventual strictures. The colonization of the peritoneum by the bacteria leads to the formation of tubercles and the thickening of the omentum [6].

Abdominal TB can be classified into four forms based on the site: peritoneal, nodal, gastrointestinal (luminal), and visceral. The most common form is luminal, with the ileocecal region being the most frequently affected site [1]. Risk factors for developing abdominal TB include underlying medical conditions such as latent TB, cirrhosis, diabetes mellitus, HIV infection, renal insufficiency, and malignancy; medical treatment with steroids and anti-tumor necrosis factor (TNF) agents; and other factors such as malnutrition, tobacco smoking, IV drug use, and alcohol consumption. However, none of these risk factors were identified in the case of the patient in question. The clinical features of abdominal TB are variable and nonspecific, ranging from fever, weight loss, night sweats, and anorexia to abdominal pain, distension, nausea, vomiting, diarrhea, and blood in the stool. Due to the wide array of symptoms, abdominal TB can mimic other diseases, such as Crohn's disease, abdominal lymphoma, and intra-abdominal malignancies. It is important to differentiate between these diseases and establish the diagnosis clearly, as the management of one could potentially aggravate the other differential, as in the case of TB and Crohn’s disease [2].

A combination of biochemical, microbiologic, and radiologic investigations may be required for diagnosis. Laboratory investigations may reveal anemia, thrombocytosis, leukocytosis, high erythrocyte sedimentation rate (ESR), high CRP, or hypoalbuminemia. Bacteriologic examination of clinical specimens such as ascitic fluid, urine, pus, or biopsy specimens through AFB smear, culture, and nucleic acid amplification test (NAAT) can help establish the definitive diagnosis of abdominal TB. While CT scan remains the superior imaging modality as it allows for the assessment of lymphadenopathy, ascites, and peritoneal and intestinal thickening, other commonly used radiologic imaging modalities include abdominal ultrasound, abdominal X-ray to identify air-fluid levels as seen in bowel obstruction, and barium enema to demonstrate mucosal ulcerations, strictures, and ileocecal valve incompetence [2]. As no pointers toward abdominal TB were present in the patient’s history, examination, and initial investigations, it was deemed appropriate to perform a CT scan to understand the pathophysiology better. The surgical team decided to move on with a diagnostic laparoscopy because the CT scan revealed indicators of a minor bowel obstruction without providing any clear indications as to the etiology.

The current World Health Organization (WHO) guidelines recommend standard antituberculous treatment (ATT) for abdominal TB, consisting of two months of treatment with isoniazid, rifampin, pyrazinamide, and ethambutol followed by four months with only isoniazid and rifampin [3]. Complete resolution with ATT is seen in most cases [7]. Surgical intervention may be required in cases of inadequate response to ATT or due to complications such as bowel obstruction, perforation, abscess, or fistula formation. A high index of suspicion is necessary for accurate diagnosis, and the relevant risk factors and presentations should be kept in mind.

A handful of cases highlight the importance of prompt diagnosis and treatment of abdominal TB, as well as the potential need for surgical intervention in some cases. The subsequent case series reports three cases of patients with abdominal TB. Two of them underwent surgical intervention, one of whom had a caecal mass causing a partial ileal obstruction, which was removed by right hemicolectomy and anastomosis, while the other two had chronic granulomatous inflammation caused by TB and underwent adhesiolysis and biopsy. All three patients were diagnosed with intestinal TB and treated with six months of ATT [8]. However, the fourth case, a 27-year-old male with suspected pulmonary TB, was diagnosed to have a bowel obstruction as a result of abdominal TB when he presented with severe abdominal pain, diarrhea, and vomiting. Unfortunately, the patient developed several complications, including sepsis, and passed away on the 18th day of hospitalization in the intensive care unit (ICU) [9].

This case highlights the importance of considering abdominal TB in the differential diagnosis of patients with unexplained abdominal symptoms. Timely diagnosis and treatment are crucial in preventing serious complications and improving patient outcomes. Healthcare providers should maintain a high index of suspicion for abdominal TB and consider it in patients with unexplained abdominal symptoms, particularly in high-risk populations.

## Conclusions

In patients from TB-endemic regions, abdominal TB should be taken into consideration as a possible differential diagnosis for bowel obstruction. Because symptoms are frequently vague and are easily confused with those of other disorders, diagnosis can be difficult. While ATT is sufficient to manage uncomplicated cases, surgical management may also be required in complicated cases that have features such as sepsis, intestinal perforation, bowel obstruction, etc.
